# Effects of socio-demographic characteristics and household water management on *Aedes aegypti* production in suburban and rural villages in Laos and Thailand

**DOI:** 10.1186/s13071-017-2107-7

**Published:** 2017-04-04

**Authors:** Nanthasane Vannavong, Razak Seidu, Thor-Axel Stenström, Nsa Dada, Hans J Overgaard

**Affiliations:** 1 Faculty of Science and Technology, Norwegian University of Life Sciences, Ås, Norway; 2 Champasak Provincial Health Office, Pakse, Lao People’s Democratic Republic; 3 Water and Environmental Engineering Group, Department of Civil Engineering, Institute for Marine Operations and Civil Engineering, Norwegian University of Science and Technology, Ålesund, Norway; 4 SARChl Chair, Institute for Water and Waste Water Technology, Durban University of Technology, Durban, South Africa; 5 Institut de Recherche pour le Développement (IRD), Maladies Infectieuses et Vecteurs, Ecologie, Génétique, Evolution et Contrôle (IRD 224-CNRS 5290 UM1-UM2), Montpellier, Cedex 5 France

**Keywords:** *Aedes aegypti*, Dengue, Laos, Thailand, Water storage

## Abstract

**Background:**

Dengue fever is a mosquito-borne disease accounting for 50–100 million annual cases globally. Laos and Thailand are countries in south-east Asia where the disease is endemic in both urban and rural areas. Household water storage containers, which are favourable breeding sites for dengue mosquitoes, are common in these areas, due to intermittent or limited access to water supply. This study assessed the effect of household water management and socio-demographic risk factors on *Aedes aegypti* infestation of water storage containers.

**Methods:**

A cross-sectional survey of 239 households in Laos (124 suburban and 115 rural), and 248 households in Thailand (127 suburban and 121 rural) was conducted. Entomological surveys alongside semi-structured interviews and observations were conducted to obtain information on *Ae. aegypti* infestation, socio-demographic factors and water management. Zero-inflated negative binomial regression models were used to assess risk factors associated with *Ae. aegypti* pupal infestation.

**Results:**

Household water management rather than socio-demographic factors were more likely to be associated with the infestation of water containers with *Ae. aegypti* pupae. Factors that was significantly associated with *Ae. aegypti* infestation were tanks, less frequent cleaning of containers, containers without lids, and containers located outdoors or in toilets/bathrooms.

**Conclusions:**

Associations between *Ae. aegypti* pupae infestation, household water management, and socio-demographic factors were found, with risk factors for *Ae. aegypti* infestation being specific to each study setting. Most of the containers did not have lids, larvicides, such as temephos was seldom used, and containers were not cleaned regularly; factors are facilitating dengue vector proliferation. It is recommended that, in Lao villages, health messages should promote proper use and maintenance of tightly fitted lids, and temephos in tanks, which were the most infested containers. Recommendations for Thailand are that small water containers should be cleaned weekly. Furthermore, in addition to health messages on dengue control provided to communities, attention should be paid to larval control for indoor containers in rural villages. Temephos or other immature control measures such as the use of pyriproxyfen, antilarval bacteria, or larvivorous fish should be used where temephos resistance is prevalent. Dengue control is not possible without additional adult mosquito control and community participation.

## Background

About 2.5 billion people are globally at risk of dengue, and 50–100 million cases of dengue fever are reported each year [1], but the number of cases is likely to be much higher [2]. The transmission of this mosquito-borne disease is considered urban, but it also occurs in rural areas [3–8]. The disease is caused by four serotypes of the dengue virus and is transmitted by two main mosquito species, *Aedes aegypti* and *Ae. albopictus* [9], which are both vectors of chikungunya and Zika viruses as well. The Lao People’s Democratic Republic (hereafter Laos) and Thailand are dengue-endemic, and all four dengue serotypes have been reported in both countries [10, 11]. Based on the national dengue surveillance data from 2006–2012 in Laos [11], one outbreak in 2010 was recorded resulting in 46 deaths. Several outbreaks have been reported in Thailand during 2000–2011, with the largest in 2010 resulting in 139 deaths [12]. A three-fold increase in the morbidity rate occurred in Laos between 2009–2010 (from 119 to 367 cases/100,000 people) while the corresponding figures for Thailand was a two-fold increase (89–184 cases/100,000 people). In the south of Laos, dengue is the most common cause of non-malaria fevers [13, 14].

Because of water scarcity, poor infrastructure and intermittent operation of water supply, the storage of water at the household level is common in many parts of the developing world, including Laos and Thailand [15, 16]. In both countries, water storage containers such as cement jars, tanks and others of various sizes have been used extensively for decades [17, 18]. Jars are normally used for storing drinking, and non-drinking water from rain and other sources piped to the house, while tanks are mostly used to store non-drinking water in toilets and bathrooms for bathing, laundry and cleaning [19]. However, as a result of improper household water management, these containers have become the preferred breeding sites for *Ae. aegypti* and an important risk factor for dengue fever transmission [17, 20–22].

Socio-demographic factors are known to affect dengue vector production and transmission. For instance, the risk of dengue in Thailand was associated with people gaining at least secondary education level and with households of more than four members [23]. Dengue modelling studies show that cases of dengue fever have a strong positive association with population density [24, 25]. Economic conditions were found to be associated with dengue cases, e.g. the seropositivity (immunoglobulin M, immunoglobulin G) of dengue was significantly associated with the absence of air-conditioning in households [26]. However, these socio-demographic factors may vary depending upon setting and other complexities of the communities like socio-economic dynamics, peoples’ knowledge and behaviour, culture and geography.

Studies on dengue risk factors associated with household water storage, management and socio-demographic characteristics have rarely been conducted, particularly in Laos. According to a previous study conducted in southern Laos and north-eastern Thailand, high values of *Stegomyia* indices and *Ae. aegypti* production in water storage containers was identified [19]. Our study was conducted to identify the risk factors of household water management and socio-demographic characteristics on *Ae. aegypti* infestation in domestic water containers. Previously selected suburban and rural villages [19], one each in Laos and Thailand, were included in this study. Results from studies like this may provide important information for *Ae. aegypti* control programs to address the increasing threat of arboviral diseases, especially in light of the recent spread of Zika outbreaks.

## Methods

### Study areas

**Fig. 1:**
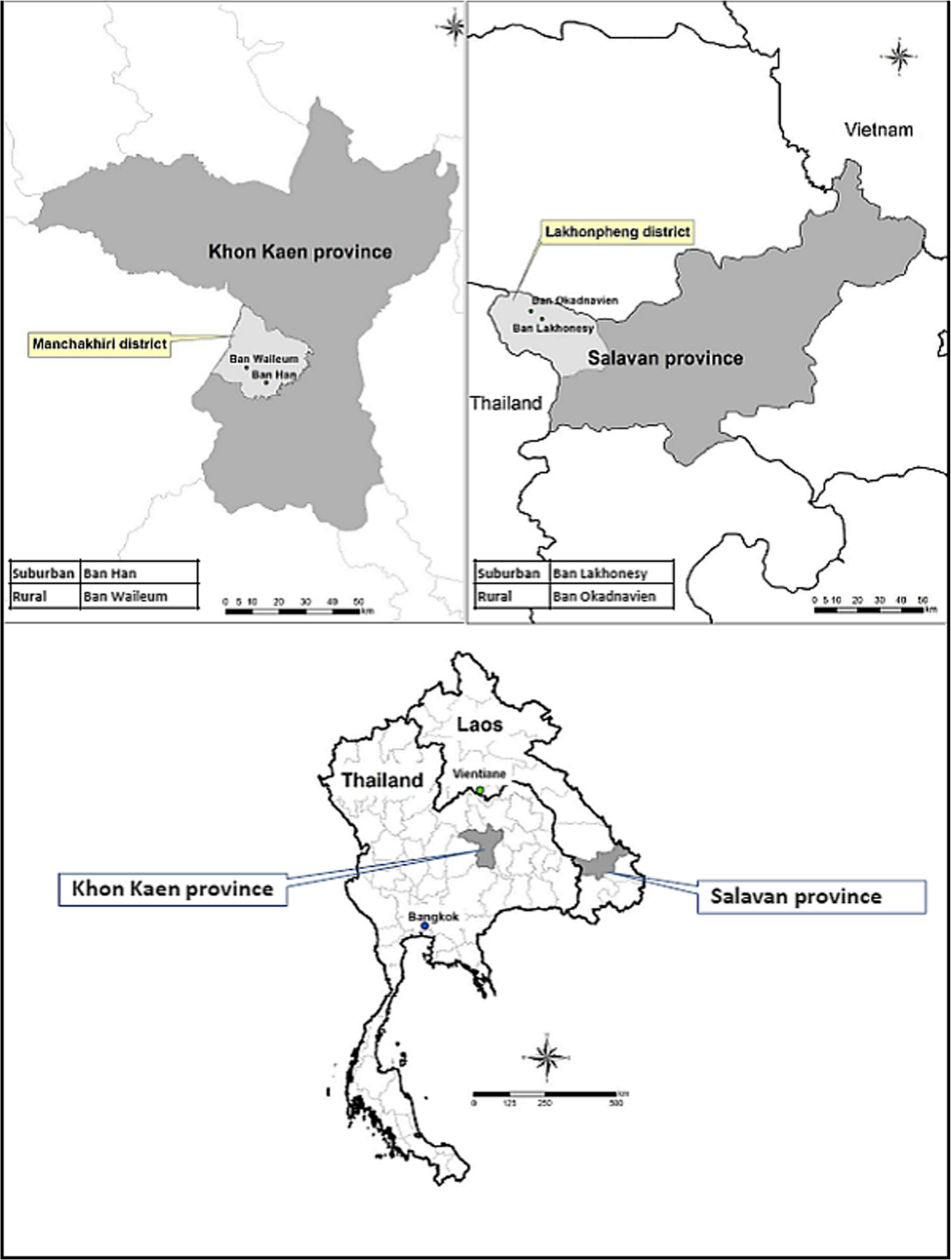
Study villages in Laos and Thailand. Reprinted from Dada et al. (2013) Relationship between *Aedes aegypti* production and occurrence of *Escherichia coli* in domestic water storage containers in rural and sub-urban villages in Thailand and Laos. *Acta Tropica*, 126:177–185, with permission from Elsevier [19]

The study was conducted from the end of February to the beginning of June 2011, corresponding to the dry to the early wet season. One suburban and one rural village each in Thailand (Feb-April) and Laos (May-June) were surveyed. The selected villages in Laos were suburban Ban Lakhonesy (15°53'29.18"N, 105°33'56.59"E) and rural Ban Okadnavien (15°55'22.37"N, 105°31'35.0"E) in Salavan province, Southern Laos. In Thailand, the villages selected were suburban Ban Han (16°07'50.71"N, 102°32'5.81"E) and rural Ban Waileum (16°10'48.95"N, 102°28'15.61"E), Khon Kaen province, northeastern Thailand (Fig. 1). The villages were selected based on previously described criteria [19].

### Study design

A cross-sectional survey of 248 households in Thailand (127 suburban and 121 rural), and 239 in Laos (124 suburban and 115 rural) was conducted. Entomological surveys alongside semi-structured interviews and observations were conducted to obtain information on *Ae. aegypti* infestation, socio-demographic factors and water management. In Thailand, the study sample represented 47 and 87% of all households in the selected suburban and rural villages, respectively. The corresponding numbers in Laos were 58% of the suburban households and 88% of the rural ones.

### Household socio-demographic characteristics

Semi-structured interviews with the heads of each selected household (respondents) were conducted in the villages. Personal information of each respondent, such as age, sex, education level and occupation, were obtained. Education was categorised into two levels: primary school or less and more education than primary school. Occupation was categorised into agriculture, commercial (e.g. shopkeepers and other business), service, and others (retired, unemployed or student). The main occupation of the people in all study villages was agriculture, especially rice farming although some people in Thailand, but not in Laos, also grow sugarcane and cassava. Information related to households’ ownership of durable assets, habitable room occupancy and access to water was also collected. In addition to the semi-structured interviews, observations were made of house material and recorded.

### Household water management and entomological survey

As part of the household water management survey, all water storage containers were classified according to type, presence or absence of a lid, the frequency of refill, and location. The sources of the household water were characterized as rain-fed (rainwater that is collected directly from the rooftop, through the roof connected tube or from a metal roofing sheet), manually collected rainwater (rainwater collected manually from larger containers), piped water into the household, or borehole water (i.e. boreholes or protected drilled wells owned by households and located in the housing areas). Containers were defined as being indoors if located under the main roof of the house or outdoors if located outside the house or under the eaves of the house. Containers in bathrooms/toilet were classified as a separate group (i.e. neither indoors nor outdoors). All household containers used for water storage were examined for mosquito pupae and larvae. If present, pupae were collected, counted, and brought back to the field station for identification using a dissecting microscope and illustrated keys as previously described [19]. All *Aedes* pupae were identified to *Ae. aegypti* and *Ae. albopictus.* Only thirteen pupae from the Lao study villages (5 suburban and 8 rural) were identified as *Ae. albopictus.* Therefore, this species was excluded in the analysis. A number of pupae were used as a dependent variable in the model of zero-inflated negative binomial regression (ZINB).

### Data analysis

Descriptive analysis of socio-demographic and household water management characteristics was conducted for each study village. Further analysis was undertaken to derive additional risk factors such as room occupancy rate and wealth status of the households. Estimation of the room occupancy rate was based on United Nation’s definition [27]. Wealth status of the households was ranked into rich, intermediate and poor using Principal Components Analysis (PCA) based on group weighted mean scores [28]. The variables used in the wealth status ranking are presented in Table 1.

**Table 1:** Variables used in the wealth status ranking

Variables	Options/Values
House material	Cement/wooden/cement-wood
House floor material	Cement/wooden/cement-wood
Room occupancy rate	> 2.5 persons per habitable room/ ≤ 2.5 persons per habitable room
Ownership of durable assets	Mobile phone/cell phone/TV/radio/refrigerator/car/motorcycle/bicycle
Affordability of bottled water	Can afford/cannot afford
Ownership of toilet facility	Yes/No
Ownership of flush toilet	Yes/No
Ownership of pour flush toilet	Yes/No

Univariate ZINB regression model was used to assess the independent effect of each of the socio-demographic and water management risk factors on the number of *Ae. aegypti* pupae in water containers. All factors included in the univariate analysis were then entered into a multivariate model to find the correlation between different factors and *Ae. aegypti* infestation in household storage water containers; and to eliminate confounding factors. The significant factors in the multivariate models were derived using backwards selection procedure. The unit of analysis with the ZINB model was the container. Statistical analyses were carried out using the statistical software SPSS 20.0 (IBM Corp.) and STATA (version 10; STATA Corporation, College Station, TX, USA). For the raw data used in the analyses please see Additional file 1.

## Results

### General information of study villages

**Fig. 2:**
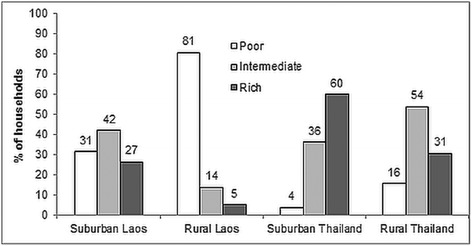
The proportion of households classified as poor, intermediate, and rich in a suburban and a rural village in Laos and Thailand. Numbers above each bar represent the percentage of households within each location

The general description of the study villages is shown in Table 2. In both rural villages, the majority of the respondents were farmers (94.8 in Laos and 95.9% in Thailand). The level of education was low in both rural villages with 95.7 and 91.7% of the respondents having no more than primary education in Laos and Thailand, respectively. In both suburban villages, on the other hand, at least 47% of respondents had at least primary education. The room occupancy rate of > 2.5 persons/habitable room was lower in Thailand compared to Laos. In Thailand, 83 and 75% in the suburban and rural village respectively had a room occupancy rate of ≤ 2.5 persons/habitable room. In Laos, this room occupancy rate was 50% in the suburban village and 63% in the rural village (Table 2). The socio-economic status (SES) was higher in Thailand compared to Laos with 60 and 31% of the households in suburban and rural Thailand falling under the rich category, respectively. Rural Laos had the highest proportion of poor households, 81% (Fig. 2).

**Table 2:** General information of respondents (household heads) and their households in a suburban and a rural village in Laos and Thailand (percentages in parentheses)

		Laos		Thailand	
		Suburban	Rural	Suburban	Rural
No. of households		124	115	127	121
Gender	Male	40 (32.3)	48 (41.7)	66 (51.9)	88 (72.7)
	Female	84 (67.7)	67 (58.3)	61 (48.1)	33 (27.3)
Occupation	Agriculture	66 (53.2)	109 (94.8)	30 (23.6)	116 (95.9)
	Commercial	12 (9.7)	3 (2.6)	34 (26.8)	1 (0.7)
	Service	30 (24.2)	3 (2.6)	11 (8.7)	2 (1.7)
	Other^a^	16 (12.9)	0	52 (40.9)	2 (1.7)
Education level	≤ Primary school	58 (46.8)	110 (95.7)	77 (60.6)	112 (92.6)
	> Primary school	66 (53.2)	5 (4.3)	50 (39.4)	9 (7.4)
Room occupancy rate	> 2.5 persons/room	62 (50.0)	43 (37.4)	22 (17.3)	30 (24.8)
	≤ 2.5 persons/room	62 (50.0)	72 (62.6)	105 (82.7)	91 (75.2)
Housing material	Cement and wood	48 (38.7)	16 (13.9)	88 (69.3)	74 (61.2)
	Cement	25 (20.2)	1 (0.9)	27 (21.3)	15 (12.4)
	Wood	51 (41.1)	98 (85.2)	12 (9.4)	32 (26.4)
Floor material	Cement and wood	29 (23.4)	14 (12.2)	82 (64.6)	62 (51.3)
	Cement	39 (31.5)	6 (5.2)	30 (23.6)	26 (21.5)
	Wood	53 (42.7)	95 (82.6)	14 (11.0)	32 (26.4)
	Ground	3 (2.4)	0	1 (0.8)	1 (0.8)

^a^Retired, unemployed and student

*Aedes aegypti* pupae positive containers were found in all four study villages (Tables 3 and 4). In Thailand, 57 and 47% of the containers were positive for pupae in the suburban and rural village, respectively. In Laos, 54% in the suburban and 33% in the rural village were pupae positive. The most important risk factors for *Ae. aegypti* pupal presence and abundance were container type (jars and tanks), location (toilets/bathrooms), lid status (no lids), education level (primary level or less, except for suburban Laos), and SES (intermediate and rich households, except in rural Laos where 81% of the households were poor). However, some factors such as water source and container cleaning frequency were site specific.

**Table 3:** Number of containers positive for *Ae. aegypti* out of those inspected and total number and density of pupae collected in relation to socio-demographic and household water management characteristics in Laos

		Suburban village				Rural village			
		Containers		Pupae		Containers		Pupae	
		No. positive/inspected	% positive	Total	No. per container (95% CI)	No. positive/inspected	% positive	Total	No. per container (95% CI)
Overall per study location		75/139	54.0	911	6.6 (4.5–8.6)	21/64	32.8	558	8.7 (2.0–15.4)
Socio-demography									
Education level	≤ Primary school	34/64	53.1	367	5.7 (3.3–8.1)	21/63	33.3	558	8.9 (2.1–15.6)
	> Primary school	41/75	54.7	544	7.2 (3.9–10.6)	0/1	0	0	–
Occupation	Agriculture	44/82	53.7	454	5.5 (3.6–7.5)	21/64	32.8	558	8.7 (2.0–15.4)
	Commercial	8/11	72.7	216	19.6 (0.3–39.0)	0	0	0	–
	Service	11/25	44.0	139	5.6 (0.9–10.2)	0	0	0	–
	Other^a^	12/21	57.1	102	4.9 (0.7–9.1)	0	0	0	–
Wealth status	Poor	21/41	51.2	240	5.9 (2.6–9.1)	19/54	35.2	514	9.5 (1.7–17.4)
	Intermediate	35/68	51.5	335	4.9 (2.8–7.1)	2/9	22.2	44	4.9 (-2.7–12.5)
	Rich	19/30	63.3	336	11.2 (3.9–18.5)	0/1	0	0	–
Household water management									
Container type	Jar	35/77	45.5	418	5.4 (2.6–8.2)	17/49	34.7	234	4.8 (1.8–7.8)
	Tank	29/41	70.7	389	9.5 (4.9–14.0)	4/6	66.7	324	54.0 (-21.6–129.6)
	Bucket	3/10	33.3	19	1.9 (-1.7–5.5)	0/7	0	0	–
Container location	Indoor	12/15	80.0	142	9.5 (3.5–15.4)	5/26	19.2	36	1.4 (-0.2–2.9)
	Outdoor	32/79	40.5	346	4.4 (1.9–6.9)	12/29	41.4	198	6.8 (1.9–11.7)
	Toilet/bathroom	31/45	68.9	423	9.4 (5.0–13.8)	4/9	44.4	324	36.0 (-12.5–84.5)
Household water source	Rain-fed	12/37	32.4	114	3.1 (0.7–5.5)	13/43	30.2	185	4.3 (0.9–7.6)
	Manually collected rain	3/3	100.0	14	4.7 (-2.9–12.3)	0/4	0	0	–
	Piped water	0	0	0	–	0	0	0	–
	Borehole	59/98	60.2	781	7.9 (5.2–10.8)	8/17	47.1	373	21.9 (-2.5–46.3)
Frequency of container cleaning	≤ Weekly	52/96	54.2	668	6.9 (4.2–9.8)	18/59	30.5	495	8.4 (1.2–15.5)
	> Weekly-monthly	22/42	52.4	238	5.7 (2.9–8.4)	3/4	75.0	63	15.8 (-12.7–44.2)
	> Monthly-yearly	1/1	100.0	5	5	0/1	0	0	–
Lid status	With lid	9/32	28.1	56	1.8 (0.0–3.5)	3/16	18.8	31	1.9 (-1.6–5.5)
	Without lid	66/107	61.7	855	7.9 (5.4–10.6)	18/48	37.5	527	10.9 (2.2–19.8)
Temephos present in container	No	53/108	49.1	668	6.2 (3.8–8.6)	19/60	31.7	446	7.4 (0.8–14.0)
	Yes	22/31	70.9	243	7.8 (3.2–12.4)	2/4	50.0	112	28.0 (-37.3–93.3)

*Abbreviation*: *CI* confidence interval

^a^Retired, unemployed and student

**Table 4:** Number of containers positive for *Ae. aegypti* out of those inspected and total number and density of pupae collected in relation to socio-demographic and household water management characteristics in Thailand

		Suburban village				Rural village			
		Containers		Pupae		Containers		Pupae	
		No. positive/inspected	% positive	Total	No. per container (95% CI)	No. positive/inspected	% positive	Total	No. per container (95% CI)
Overall per study location		97/171	56.7	1,046	6.1 (3.2–9.0)	85/179	47.5	1,007	5.6 (2.9–8.3)
Socio-demography									
Education level	≤ Primary school	71/115	61.7	833	7.2 (3.2–11.3)	72/161	44.7	658	4.1 (2.0–6.1)
	> Primary school	26/56	46.4	213	3.8 (1.1–6.6)	13/18	72.2	349	19.4 (-0.4–39.2)
Occupation	Agriculture	21/35	60.0	115	3.3 (1.3–5.3)	79/168	47.0	962	5.7 (2.9–8.6)
	Commercial	23/34	67.6	229	6.7 (1.4–12.1)	2/3	66.7	4	1.3 (-1.5–4.2)
	Service	5/13	38.5	8	0.6 (0.1–1.2)	2/3	66.7	13	4.3 (-8.4–17.1)
	Other^a^	48/89	53.9	694	7.8 (2.7–12.9)	2/5	40.0	28	5.6 (-7.9–19.2)
Wealth status	Poor	4/8	50.0	47	5.9 (-5.1–16.9)	8/19	42.1	30	1.6 (0.4–2.8)
	Intermediate	35/64	54.7	574	8.9 (1.9–15.9)	39/101	38.6	534	5.3 (1.7–8.9)
	Rich	58/99	58.6	425	4.3 (2.2–6.3)	38/59	64.4	443	7.5 (2.1–12.9)
Household water management									
Container type	Jar	38/69	55.1	370	5.4 (0.5–10.2)	41/100	41.0	429	4.3 (0.7–7.8)
	Tank	51/85	60.0	595	7.0 (2.9–11.1)	42/74	56.8	572	7.7 (3.3–12.1)
	Bucket	8/17	47.1	81	4.8 (-3.4–12.9)	2/5	40.0	6	1.2 (-0.8–3.2)
Container location	Indoor	24/44	54.5	106	2.4 (1.2–3.6)	18/56	32.1	335	5.9 (-0.3–12.3)
	Outdoor	11/20	55.0	247	12.4 (-4.8–29.5)	15/35	42.9	91	2.6 (0.6–4.6)
	Toilet/bathroom	62/107	57.9	693	6.5 (3.0–9.9)	52/88	59.1	581	6.6 (2.9–10.3)
Household water source	Rain-fed	8/15	53.3	15	1.0 (0.1–1.9)	4/12	33.3	37	3.1 (-0.6–6.7)
	Manually collected rain	2/6	33.3	2	0.3 (-0.2–0.9)	9/34	26.5	120	3.5 (-1.9–8.9)
	Piped water	84/145	57.9	1,022	7.1 (3.7–10.4)	70/131	53.4	848	6.5 (3.1–9.9)
	Borehole	0	0	0	–	0	0	0	–
Frequency of container cleaning	≤ Weekly	37/71	52.1	168	2.4 (1.3–3.5)	27/60	45.0	146	2.4 (1.2–3.7)
	> Weekly-monthly	55/92	59.8	864	9.4 (4.1–14.6)	55/111	49.5	828	7.5 (3.2–11.7)
	> Monthly-yearly	5/8	62.5	14	1.8 (-0.2–3.7)	3/8	37.5	33	4.1 (-2.5–10.8)
Lid status	With lid	13/29	44.8	24	0.8 (0.2–1.5)	12/45	26.7	145	3.2 (-0.9–7.3)
	Without lid	84/142	59.2	1,022	7.2 (3.7–10.6)	73/134	54.5	862	6.4 (3.1–9.7)
Temephos present in container	No	97/171	56.7	1,046	6.1 (3.2–9.0)	83/177	46.9	983	5.5 (2.9–8.3)
	Yes	0	0	0	–	2/2	100.0	24	12.0 (-51.5–75.5)

*Abbreviation: CI* confidence interval

^a^Retired, unemployed and student

### Effect of socio-demographic characteristics on *Aedes aegypti* production

The univariate analysis (Table 5) showed that households in suburban Laos where the respondent’s occupation was ‘commercial’ were significantly associated with *Ae. aegypti* pupae abundance (IRR 2.9, 95% CI: 1.01–8.8) compared to agricultural households. In suburban Thailand, respondents involved in ‘other’ occupations (retired, unemployed or student), were about three times more likely to have *Ae. aegypti* pupae in their homes, whereas, those who were services were less likely to have *Ae. aegypti* in their homes (IRR 0.2, 95% CI: 0.1–0.8) compared to farmers’ households. In the multivariate analysis, only ‘other’ occupations (IRR 2.3, 95% CI: 1.1–4.8) in suburban Thailand remained significantly associated with *Ae. aegypti* (Table 6). In rural Thailand, ‘commercial’ occupations were less likely to have *Ae. aegypti* infestation in their homes (IRR 0.1, 95% CI: 0.01–0.8) compared to those with agriculture occupation (Table 6). In rural Thailand, the houses of respondents who had a higher education than primary school were four times more likely to be infested with *Ae. aegypti* than in houses of respondents with lower education (Table 5). Households in rural Thailand assessed as being intermediate or rich were each about five times more likely to have their homes infested with *Ae. aegypti* compared to poor households (Table 5). In the multivariate model, these relationships became much stronger with households in the intermediate (IRR 9.3, 95% CI: 3.1–28.1) and rich SES categories (IRR 13.2, 95% CI: 4.01–43.3) being significantly associated with *Ae. aegypti* (Table 6).

**Table 5:** Incidence rate ratios, IRR (95% confidence intervals) by univariate analysis of *Ae. aegypti* pupae per container in relation to socio-demographic and household water management in a suburban and a rural village in Laos and Thailand

		Laos				Thailand			
		Suburban		Rural		Suburban		Rural	
No. of containers		139		64		171		179	
Socio-demography									
Education level	≤ Primary school	64	1	63	1	115	1	161	1
	> Primary school	75	1.3 (0.6–2.6)	1	na	56	0.6 (0.3–1.2)	18	4.1 (1.4–12.2)**
Occupation	Agriculture	82	1	64	1	35	1	168	1
	Commercial	11	2.9 (1.0–8.8)*	0	na	34	2.1 (0.8–5.1)	3	na
	Service	25	1.3 (0.5–3.3)	0	na	13	0.2 (0.1–0.8)*	3	na
	Other^a^	21	0.8 (0.3–1.9)	0	na	89	2.8 (1.2–6.3)*	5	na
Wealth status	Poor	41	1	54	1	8	1	19	1
	Intermediate	68	0.8 (0.4–1.8)	9	na	64	1.5 (0.3–8.6)	101	5.2 (1.8–15.2)**
	Rich	30	1.7 (0.7–4.2)	1	na	99	0.6 (0.1–3.5)	59	4.8 (1.7–13.6)**
Household water management									
Container type and location									
Jar	No	62	1	15	1	102	1	79	1
	Yes	77	0.9 (0.5–1.9)	49	0.2 (0.1–0.5)**	69	0.8 (0.4–1.5)	100	0.7 (0.3–1.5)
Tank	No	98	1	58	1	86	1	105	1
	Yes	41	1.2 (0.6–2.5)	6	6.3 (2.0–19.9)**	85	1.3 (0.7–2.5)	74	1.5 (0.7–3.1)
Bucket	No	129	1	57	1	154	1	174	1
	Yes	10	0.4 (0.1–2.5)	7	na	17	0.8 (0.2–2.9)	5	0.2 (0.0–1.9)
Container location	Indoor	15	1	26	1	44	1	56	1
	Outdoor	79	0.8 (0.3–2.1)	29	2.5 (0.8–7.4)	20	5.9 (1.8–19.9)**	35	0.2 (0.1–0.8)*
	Toilet/bathroom	45	1.1 (0.4–2.8)	9	12.8 (3.4–48.6)**	107	2.7 (1.3–5.6)**	88	0.6 (0.2–1.4)
Water source, cleaning and lids									
Rain-fed	No	102	1	21	1	156	1	167	1
	Yes	37	0.7 (0.3–1.8)	43	0.3 (0.1–0.9)*	15	na	12	0.7 (0.1–4.3)
Manually collected rain	No	136	1	60	1	165	1	145	1
	Yes	3	0.5 (0.1–3.1)	4	na	6	0.1 (0.0–0.5)**	34	1.2 (0.3–3.9)
Piped water	No	0		0		26	1	48	1
	Yes	0		0		145	na	131	1.2 (0.5–3.2)
Borehole	No	41	1	47	1	0		0	
	Yes	98	1.8 (0.8–4.2)	17	3.6 (1.2–11.1)*	0		0	
Frequency of container cleaning	≤ Weekly	96	1	59	1	71	1	60	1
	> Weekly-monthly	42	0.8 (0.4–1.7)	4	na	92	4.2 (2.1–8.2)**	111	3.5 (1.6–7.4)**
	> Monthly-yearly	1	0.5 (0.0–14.7)	1	na	8	0.7 (0.2–3.3)	8	2.4 (0.3–18.6)
Lid status	Without lid	107	1	48	1	142	1	134	1
	With lid	32	0.4 (0.1–1.1)	16	0.3 (0.1–1.8)	29	0.1 (0.0–0.3)**	45	0.9 (0.3–2.9)
Temephos present in container	No	108	1	60	1	171	1	177	1
	Yes	3	0.9 (0.4–1.8)	4	2.6 (0.3–21.5)	0	na	2	2.2 (0.1–56.2)

*P ≤ 0.05, **P ≤ 0.01

^a^Retired, unemployed and student

*Abbreviation*: *na* not applicable

**Table 6:** Incidence rate ratios, IRR (95% confidence intervals) by multivariate analysis of *Ae. aegypti* pupae per container in relation to socio-demographic and household water management in a suburban and a rural village in Laos and Thailand

		Laos				Thailand			
		Suburban		Rural		Suburban		Rural	
No. of containers		139		64		171		179	
Socio-demography									
Occupation	Agriculture					35	1	168	1
	Commercial					34	1.9 (0.8–4.9)	3	0.1 (0.0–0.8)*
	Service					13	0.6 (0.1–3.2)	3	0.9 (0.1–12.5)
	Other^a^					89	2.3 (1.1–4.8)*	5	1.5 (0.2–9.3)
Wealth status	Poor							19	1
	Intermediate							101	9.3 (3.1–28.1)**
	Rich							59	13.2 (4.0–43.3)**
Household water management									
Tank	No			58	1				
	Yes			6	5.9 (1.9–19.1)**				
Container location	Indoor	15	1					56	1
	Outdoor	79	0.6 (0.2–1.6)					35	0.2 (0.1–0.5)**
	Toilet/bathroom	45	0.7 (0.2–2.1)					88	0.4 (0.2–0.9)*
Frequency of container cleaning	≤ Weekly					71	1	60	1
	> Weekly-monthly					92	3.5 (1.9–6.6)**	111	2.6 (1.3–5.1)**
	> Monthly-yearly					8	2.4 (0.5–12.2)	8	5.9 (0.6-55.2)
Lid status	Without lid	107	1	48		142	1		
	With lid	32	0.3 (0.1–0.9)*	16	0.7 (0.2–2.6)	29	0.1 (0.0–0.4)**		

*P ≤ 0.05, **P ≤ 0.01

^a^Retired, unemployed and student

### Effect of household water management on *Aedes aegypti* production

#### Container types and locations

Jars and tanks were the most commonly used water storage containers across all four villages (Tables 3 and 4). The univariate model showed that container type was only significantly associated with *Ae. aegypti* pupae in rural Laos, and not in any other study village. Here, jars were the least likely to be infested (IRR 0.2, 95% CI: 0.1–0.5) when compared to non-jar containers, and tanks were the most likely to be infested (IRR 6.3, 95% CI: 2.0–19.9) when compared to non-tanks (Table 5). In the multivariate analysis, tanks remained the most likely to be infested (IRR 5.9, 95% CI: 1.9–19.1), while jars were not significant (Table 6).

In Laos, 57% of water storage containers in the suburban village and 45% in the rural village were located outdoors. In Thailand, 63% of the containers in the suburban village and 49% in the rural village were found in toilets or bathrooms (Tables 3 and 4). For rural Laos, the univariate model showed that containers located in the toilet/bathroom were about 13 times more likely to be infested with *Ae. aegypti* pupae than those located indoors (Table 5). Container location was not of importance in suburban Laos. In suburban Thailand, containers located in the toilet or bathroom (IRR 2.7, 95% CI: 1.3–5.6) and those located outdoors (IRR 5.9, 95% CI: 1.8–19.9) were more likely to be infested than indoor containers (Table 5). However, in rural Thailand, the opposite was observed; containers located outdoors were less likely to be infested compared to those located indoors (IRR 0.2, 95% CI: 0.1–0.8) (Table 5). In the multivariate model for rural Thailand, containers located outdoors (IRR 0.2, 95% CI: 0.1–0.5) and in the toilet/bathroom (IRR 0.4, 95% CI: 0.2–0.9) were significant less likely to be associated with *Ae. aegypti* pupae infestation (Table 6).

#### Water sources

In rural Laos, the univariate analysis showed that containers with water from boreholes and rainwater were significantly associated with *Ae. aegypti*. Containers with borehole water were 3.6 times (IRR 3.6, 95% CI: 1.2–11.1) more likely to be infested than containers with non-borehole water. Rain-fed water was significantly less likely to be infested with *Ae. aegypti* pupae than containers with other water sources (IRR 0.3, 95% CI: 0.1–0.9). Similar outcomes were obtained in suburban Thailand, with manually collected rainwater being less likely to be infested (IRR 0.1, 95% CI: 0.01–0.5) when other water sources were used as a reference. None of the water sources recorded was significantly associated with *Ae. aegypti* pupae in suburban Laos and rural Thailand. The multivariate model did not show any significant associations between water source and *Ae. aegypti* pupae across all four villages.

#### Frequency of container cleaning, lid status and a presence of temephos in container

In Laos, most of the containers in both rural (92%) and suburban (69%) villages were cleaned every week. The frequency of cleaning was not significantly associated with *Ae. aegypti*. In Thailand, the majority of the containers were cleaned less often than those in Laos. Fifty-four percent and 62% of the containers in suburban and rural Thailand, respectively, were cleaned once during a period of a week and up to one month (Tables 3 and 4). As a result, these containers were more likely to be associated with *Ae. aegypti* in both the suburban (IRR 4.2, 95% CI: 2.1–8.2) and the rural (IRR 3.5, 95% CI: 1.6–7.4) villages (univariate model) compared to containers that were cleaned once a week (Table 5). This association remained significant in the multivariate model in the suburban (IRR 3.5, 95% CI: 1.9–6.6) and the rural (IRR 2.6, 95% CI: 1.3–5.1) village, respectively (Table 6).

In all study villages, most of the containers did not have lids. Only 23 and 25% of those in suburban and rural Laos, and 17 and 25% in suburban and rural Thailand, respectively were covered. Containers with lids were significantly less likely to be infested than those without lids in the suburban villages in Laos (IRR 0.3, 95% CI: 0.1–0.9) and Thailand (IRR 0.1, 95% CI: 0.04–0.4) (Table 6). None of these associations was significant in the rural villages in either country (Tables 5 and 6).

In Laos, 22% of water storage containers in the suburban village and 6% in the rural village used the larvicide temephos (Abate). In Thailand, only one percent of containers in the rural village had temephos (Tables 3 and 4). There were no significant associations between temephos and *Ae. aegypti* pupae in both the univariate and multivariate models (Tables 5 and 6).

## Discussion

The relationships between mosquito breeding and socio-economic and water management factors are complex as shown in the study. Several risk factors associated with *Ae. aegypti* pupae infestation were relatively site specific. In Thailand, both socio-demographic and water management factors were to different degrees related to immature *Ae. aegypti* production. Very few significant associations between immature *Ae. aegypti* production and socio-demographic and water management factors were found in suburban Laos. The rural village is excluded from comparisons since it was a homogenous poor low-educational agricultural community.

### Socio-demographic relationships

Specifically, the occupation of the household head and household wealth status were significantly associated with *Ae. aegypti* infestation in Thailand. The significance of occupation varied and was site specific. In suburban Laos where the respondent’s occupation was ‘commercial’, there were significant associations with *Ae. aegypti* pupae abundance (IRR 2.9, 95% CI: 1.01–8.8) compared to agricultural households (which was the reference). In suburban Thailand, significant associations were also found with *Ae. aegypti* infestation, but in households where the occupation of the household head was ‘other’ (retired, unemployed or student). Households with ‘other’ occupations are not economically active and were the largest group (41%) in the suburban village. Another study also showed that non-economically active people were about 1.6 times more likely to have their households present with *Ae. aegypti* [29]. Those with agricultural occupations had a lower likelihood in both suburban sites.

With regards to household wealth status in the rural Thai village, the intermediate households were nine times, and the rich households 13 times, more likely to have their home water containers infested with *Ae. aegypti* compared to the poorer households (Table 6). This may be because the intermediate and rich households had more water containers than the poor ones, thereby providing more breeding sites for *Ae. aegypti*. This contrasts a previous study in Colombian towns where water containers in rich households were less likely to be infested with *Ae. aegypti* immatures compared to poor households [30].

### Water management factors

Cement tanks were significantly more likely to be infested with *Ae. aegypti* compared to other containers in Laos (Tables 5 and 6). In both the suburban and the rural villages, cement tanks without lids were used to store non-drinking water in the toilets or bathrooms. The major challenge for *Ae. aegypti* larval control in many countries in south-east Asia is that such tanks, which are difficult to cover, are more likely to be used on a large scale [19, 21, 31]. In addition, large containers are often difficult to clean more frequently to enable the interruption and prevention of mosquito life-cycle and mosquito production. Less frequent cleaning provides good breeding sites for dengue vector production [32–36]. It is thus not the container type as such that is a factor for consideration but rather the combination of container size, their placement, no or poorly fitted lids and low frequency of cleaning that is the combined determinant of *Aedes* infestation. This is further supported by the other types of containers, such as jars in Laos, which were significantly less likely to be infested with *Ae. aegypti,* especially in the rural village (Table 5) possibly due to their predominant use for the storage of drinking water and hence better handling (e.g. use of lids) and hygiene. This finding was contrary to those made in other studies where jars were considered a high-risk factor for mosquito breeding in Laos [37] and in Thailand [22], but again the combined purpose and handling practices will play a major role.

In the suburban village in Thailand, containers located outdoors and in toilets or bathrooms were more likely to be infested with *Ae. aegypti* than those located indoors, but in the rural village, outdoor containers were less likely to be infested (Table 5). It is unclear why this is so, but this could be because indoor containers in the suburban households were better handled and more often had lids than in the rural households. Rural households may provide better access for mosquitoes to indoor containers, which would be located in dark spaces, not well protected with lids and potentially providing attractive breeding sites. Again it is not only partly attributed to handling practices but also the purpose of the containers (example for drinking where the handling care is higher), our data showed there were more drinking water containers located outdoors in rural than in suburban villages of Thailand. In the suburban village outdoor containers were less often used for drinking (i.e. poorer hygiene). Other studies have shown that containers located outdoors are the main dengue vector producers compared to those located indoors [32, 34, 35, 38]. However, in Vietnam, the majority of *Ae. aegypti* immatures was found in indoor containers rather than outdoor containers [31, 39]. Our results show the complexity of *Ae. aegypti* breeding, as they breed in a wide range of *household containers* regardless of location, especially under similar environmental conditions.

The handling practices are further supported by the cleaning practises and container type. Containers in Thailand were less frequently cleaned than those in Laos, due to a higher frequency of large containers in Thailand (e.g. large cement jar containing up to 2,000 l of water). Containers cleaned on a weekly basis were less likely to be infested with *Ae. aegypti*. A weekly cleaning schedule is also recommended by many national public health authorities and by WHO [40, 41]. The effectiveness of frequent cleaning has also been confirmed by studies in northern Thailand [33] and in six other Asian countries [35].

This study also showed that containers with lids act as prevention against mosquito breeding. Containers with lids were significantly less likely to be infested with *Ae. aegypti* compared to those without lids in Laos (Table 6). This has also been shown in many other studies where containers without lids or partly covered produced more *Ae. aegypti* than those with lids [16, 31, 37, 42]. Container lids are not an absolute barrier and must be tightly fitted to prevent gravid females to enter for oviposition [43, 44]. Such lids are a low-cost, effective and environmental friendly intervention for dengue vector control and have also been recommended by the WHO [40]. However, this intervention needs to be properly managed and maintained by communities.

In rural Laos, containers with borehole water were almost four times more likely to be infested with *Ae. aegypti* pupae compared to containers with other water sources (Table 5). Many containers with borehole water (53%) (data not presented in results) were located in toilets or bathrooms, and all of them were without lids, all conditions that provide good breeding sites for *Ae. aegypti* [21, 31]. Conversely, rain-fed water was less likely to be infested compared to other water sources (Table 5). This could be because as many as 75% (data not presented in results) of the rain-fed containers were covered with lids and used for drinking. However, in the multivariate analysis (Table 6), water sources were not significantly associated with *Ae. aegypti* infestation.

Laos and Thailand have similar dengue outbreak responses. The so-called Surveillance and Rapid Response Teams (SRRT) act rapidly, within 24 h when a dengue case is diagnosed by a physician, to implement vector control measures. Such measures usually consist of space spraying with thermal fog within a radius of 100 m from the affected house. In addition, the larvicide temephos (Abate® 1% sand granules) is freely provided nationwide in both countries. A systematic literature review showed that temephos was effective against *Ae. aegypti* production in water storage containers [45]. In the present study, it was found that some water containers in both villages of Laos contained temephos, but was mainly absent in Thailand (Tables 3 and 4). Thus, temephos was inconsistently used and may not have been an effective dengue control intervention in these settings. This could be due to problems of distribution or perceptions of temephos as a harmful chemical as well as improper use, which has been described in a study conducted in northeastern Thailand [46]. The indiscriminate use of temephos can lead to temephos resistance as identified in some parts of Thailand [47, 48]. Pyriproxyfen, spinosad, or antilarval bacteria (e.g. *Bti*) have been shown to be effective against *Ae. aegypti* [49] and could be used instead of temephos. Control measures such as the use of larvivorous fishes were not observed in the examined containers but were sometimes observed in containers in other households not included in the study. Personal protection using repellents to control adult mosquitoes was also observed, but not accounted for in this study. However, for any of these control measures to be effective against *Ae. aegypti*, the involvement of multi-sectoral stakeholders as well as active community participation is key [50].

This study was a cross-sectional survey carried out at the end of the dry and the beginning of the wet season. Risk factors related to household water management may vary between seasons, and between years. The number of rainwater storage containers in the wet season would be higher than observed in our study, providing more breeding sites. Also, our study might have influenced nearby households to take action to clean out positive mosquito containers in their homes and thus biasing the results.

## Conclusions

This study showed a relationship between *Ae. aegypti* production in water storage containers and risk factors associated with socio-demography and households’ water management practices. Most of the risk factors were specific to the study villages. Our study showed that household water management rather than socio-demographic factors were more likely to be associated with the infestation of water containers with *Ae. aegypti*. Most of the containers did not have lids, were not protected with larvicides and were not cleaned regularly, thereby providing breeding sites for dengue vectors. As the aforementioned risk factors were significantly associated with *Ae. aegypti* infestation, it is recommended that, in Lao villages, health messages should promote proper use and maintenance of tightly fitted lids, and temephos in tanks, which were the most infested containers. Recommendations for Thailand are that small water containers should be cleaned weekly. Furthermore, attention should be paid to larval control for indoor containers in a rural village in addition to health messages. Temephos, which is the first larval control method of choice today, can be used in areas without temephos resistance. Alternatively, pyriproxyfen, spinosad, antilarval bacteria (e.g. *Bti*) or larvivorous fish should be considered where temephos resistance is prevalent. However, adult mosquito control must also be considered in an integrated vector management strategy. Compliance is always an issue when it comes to mosquito control. Therefore, community participation will be key to the success of any selected control measure.

## Abbreviations

IRR: Incidence rate ratios
PCA: Principal Components Analysis
SES: Socioeconomic status
SRRT: Surveillance and Rapid Response Teams
UN: United Nations
WHO: World Health Organization
ZINB: Zero-inflated negative binomial
